# Supplementary material for: Smart deworming collar: A novel tool for reducing Echinococcus infection in dogs
Source: PLoS Negl Trop Dis. 2021 Jul 8;15(7):e0009443. doi: 10.1371/journal.pntd.0009443 (PMC8266112; doi:10.1371/journal.pntd.0009443)
Supplement: Attachment — Submitted filename: A letter responses to the review comments1.docx [file pntd.0009443.s002.docx]

**A point-to-point response letter**

Dear Editor,

Thank you very much for the professional opinions and suggestions. According to these professional opinions and Manuscript Body Formatting Guidelines of PLOS Neglected Tropical Diseases, we performed the resubmission of a significantly-revised version of our original manuscript, in which we increased page numbers and line numbers, adjusted the line to spacing 2 times, added 3 references and adjusted the order of all references, re-describled the statistical analysis methods and results, used the Preflight Analysis and Conversion Engine (PACE) digital diagnostic tool to make Fig 1 and 2 meet the requirements of PLOS Neglected Tropical Diseases, added, improved and optimized related tables, added a lot of details about the content that editor teachers and reviewers concerned.

We are looking forward to seeing this manuscript could be published soon.

Best,

Xiao-Nong Zhou, Corresponding author on behalf of all co-authors.

For more details, please see the revised manuscript and the reply letter containing a detailed list of our responses to the review comments as follows:

| Category | Reviewer | Specific questions and suggestions | Response to many specific questions and suggestions |
| --- | --- | --- | --- |
| Methods | Reviewer #1 | Prior to describing the appearance of the device, it would be helpful to describe briefly the purpose of the collar and how it is used.  Are the praziquantel baits flavored?  Are dogs supposed to notice that a bait has fallen out of the collar and eat it without human assistance?  How can one confirm that the bait was actually eaten by the dog wearing the collar, especially if the dog is moving when the bait drops and/or a human isn’t present? | Thank you very much for good professional advice. we briefly describe the purpose of the collar and how it is used in the part of Interventions of Methods in revised manuscript, and we added the reference 25, in which we described in details the functions and advantages of the smart deworming collar as well as its installation and using steps  The steps for attaching the smart collar to the dogs were as follows: (1) matching the information of the dogs, the owners, and the smart collars and uploading the information to the RMS; (2) set deworming frequency such as once a month, once a quarter, even once every six months according to the local prevalence of echinococcosis; (3) set the PZQ baits dose for each dog according to the dog’s weight and size; and (4) set deworming time (6:00 a.m.on the deworming date), and then the smart deworming collar will run according to the set procedure.  As you mentioned, the PZQ baits themselves also have a tempting flavor to attract dogs eating.  In order to attract the attention of dogs, we have done a lot of meticulous works, including the deworming reminder function set in the smart deworming collar, the choice of the deworming time(at 6 AM on the morning of every month’s deworming day, when most dogs are still sleeping (not run or walk), and the tempting bait. The detailed measures described in the part of Interventions of revised manuscript to try our best to let the dog notice the bait, find the bait and eat the bait. Despite this, we do not still ensure that every dog can find the bait and eat it by itself. In our study described in Table 5, the PZQ-swallowing rate was 58.2% (in 10 min), and 71.2% (in 30 min) respectively. In fact, It is our goals that manual delivery can be replaced by smart delivery for every dog to increase the deworming coverage and frequency as well as active swallowing rate in the future and we will continue to work hard for it. Thank you very much again. |
|  |  | Are both Seni and Hezuo pastoralist communities? What is the approximate size of these communities? | Yes, both of them are pastoralist communities with about 144,000 and 95,000 people respectively, and we added the information in Field settings of Methods in revised manuscript. |
|  |  | Please briefly describe the registration methods/requirements for dogs in the study area. Were there any inclusion/exclusion criteria for enrollment in this study (e.g., size/weight of the dog)? | Thank you very much for your suggestions. The requirements for dogs, and the inclusion and exclusion criteria of dogs are very important and we have added this in the part of Grouping and sample size in revised manuscript in accordance with your suggestions. The method and steps of dog registration described in the part of Interventions of Methods in revised manuscript. For more details, please see the references 25 and 26. |
|  |  | Briefly provide the instructions given to the owners of dogs in the collar and non-collar groups regarding deworming, record keeping, etc. It was not clear if owners in the manual deworming group were given instructions to deworm the dog or if a local veterinarian was responsible for dog deworming and, if both occurred, how the analysis took this into account. | Thank you very much for your suggestions. In the smart deworming collar group, the deworming information including deworming reminder, deworming time and times were be recorded and uploaded onto the RMS automatically. The delivery and deworming implemented by the smart collars controlled by RMS. In manual deworming group, the dog information, The dog owner’s information were entered into the RMS by the local veterinarians, the baits distribution is completed by the local veterinarians, and the deworming works and records are completed by the dog owners. Detailed modification was in the part of Interventions of revised manuscript. |
|  |  | Please elaborate on what dog and owner information was initially collected and uploaded to the RMS. Please also clearly provide a description of what data the collar collects and how this information is downloaded from the collar (and the frequency at which it is downloaded). | Dog information includes the dog’s gender, age, color, breed, weight, size and the name of its owner, etc. The dog owner’s information includes gender, age, ID number, mobile phone number, ethnicity, production and lifestyle, occupation, and village name, etc. The smart deworming collar information includes the collar ID number, manufacturer, etc. The above information, together with the deworming time and the deworming dosage, matched and then uploaded them to the RMS automatically when the smart deworming collar first attached for dogs. When the smart deworming collar starts to work, it will automatically collect information including the environment temperature, its battery power, deworming reminder, deworming times, deworming failures, etc., when the managers login the RMS, all information will be displayed, statistically analyzed, and download. For more details about all the information and the RMS, please see reference 26. |
|  |  | Is there a published reference for the copro-ELISA’s sensitivity and specificity? | Yes, there is a published reference for the copro-ELISA’s sensitivity and specificity. please see https://kns.cnki.net/kcms/detail/detail.aspx?dbcode=CJFD&dbname=CJFDLAST2020&filename=HBYS202009007&v=BIIMyk7mNQe3Wkx%25mmd2BXJl9DgHydP2LteLDKVXplO0o4%25mmd2F2FryCt5n5rGQmy0Ov9bpEr. |
|  |  | Please elaborate on when the questionnaire was administered. The paper states that the questionnaire was completed by the dog’s owner at the beginning of the study. However, some of the questions appear to be addressing collar compliance issues, which would not be available immediately. Was a questionnaire also administered to the control group? | Thank you very much for your questions. The questionnaires was conducted twice on the dog owners in the beginning and end of study respectively, and the subject of the questionnaire survey is the owner of the dog wearing the smart collar, so the questionnaire was only administered in the smart deworming collar group. |
|  |  | Please elaborate on the statistical methods used, including what specific variables were assessed. The statement, “…(GEE) was employed to analyze the data which were the categorical outcome variables obtained by continuous repeated measures” is not clear. | Thank you very much for your suggestions. We re-describe the statistical methods used in order to show our statistical methods, specific variables, and results more clearly. Detailed modification was in the part of Data analysis in Methods and the Result in revised manuscript. |
|  |  | Much of the information located in the footnotes of tables 2-4 seems important enough to be included in the Methods text. | Thank you very much for your suggestions. The information located in the footnotes of tables 2-4 is important enough, according to your suggestions, we removed these contents to the appropriate places in the method section. Furthermore, we rearranged (merged, added, revised) the tables, and revised the number of the table, please see more details of the modification in our revised manuscript. |
|  | Reviewer #2: | the work is clear, the objectives are well planned and the development is well articulated | Thank you very much for your encouragement and recognition of our work. We know that although our work is meaningful, there are still many limitations. We will continue to work hard to do our best. Thanks again. |
| Results | Reviewer #1 | What is meant by the “dogs’ incompatibility with the collar’? | Thank you very much for your question. In order to make the dog wearing a smart deworming collar comfortable, the weight and size of the collar have certain restrictions (please see Design of the deworming device of Methods in revised manuscript). The dog which size is too large or which weight is less than 5kg or which age is less than 1 months is not suitable for wearing smart deworming collars, that is, some smart deworming collars not match (are incompatible) with too large dogs or too small dogs (please see Grouping and sample size of Methods in revised manuscript). In fact, most dogs are suitable (matched or compatible) for wearing the smart deworming collars. We speculate that it was the dog owner themselves who refused to wear collars for dogs by some other reason. |
|  |  | What happened to dogs whose owners elected to remove the collar during the study period (e.g., if an owner removed the collar at 6 months, were they given instructions to manually deworm the dog for the remainder of the study)? | Thank you very much. The questions are very important. When the owner removed the collar during the study period, we had not given instructions to deworm the dog manually. We regard this phenomenon as a withdrawal halfway of the study. Moreover, in order to eliminate the deviation and better evaluate the effect of the smart collar, whether to deworm for the dog or not decided by the dog owner, just like the practice in the manual deworming group. On the other hand, the related researches of the dogs such as feces detection and observation and recording of the collars continued. |
|  |  | Table 1- Please explain the missing numbers column. Were there any dogs lost to follow-up during the study? | Thank you very much for your question. Due to pastures’ dog-raising habits, and production lifestyles such as the transition between the pastures (winter) and the ranch (summer), the range of movement of herding dogs is very large, it is normal to be lost to follow-up at a certain time-point for collecting dog feces. In fact, the missing numbers of the dog at a certain time to collect dog feces is the dog numbers lost to follow-up at the time. A dog lost in this follow-up time (time to collect dog feces) does not mean it will be lost forever, in fact, most of them appeared in the next follow-up time (time to collect dog feces). |
|  |  | Table 4- How can the authors be sure that the dogs actually ingested the praziquantel in 10 minutes or 30 minutes?  Also, what is meant be “dogs’ reaction rate”?  What is a deworming reminder? | Thank you very much for the questions. The staff observed the first deworming process of a smart collar to confirm that it worked normally, record the information dogs swallowed PQZ baits (30 minutes), and then, during the questionnaire survey, the local veterinarian asked the dog owners about the PQZ baits swallowed.  In addition, the dog reactions to the collar attachment means (1) moved in circles, (2) trying to bite the collar, (3) remove the collar by themselves when the smart collar attached to the dog. The deworming reminder is a basic function of the smart collar to wake up the dog when it is time to deliver the PQZ bait for swallowing. The sound of deworming reminder lasts for three minutes before the smart collar deliver the PQZ bait. We have added details about the dogs’ reaction and deworming reminder in the part of Result in revised manuscript. |
|  |  | Can you provide additional information about the dogs that became infected during the study? Did you find new dogs infected at each sampling or were there dogs that were positive over multiple time points? | Thank you very much for your professional question. In the smart collar group, whether in Seni or Hezuo, most of the positive dogs are the same dog before and after the deworming. One of dogs in Seni dewormed after the sixth was newly infected, and so did the dog that dewormed for the ninth time in Hezuo. In the control group, we found many dogs were positive at multiple times. Of course, there were also new infected dogs. This is very interesting, we will analyze it in other manuscripts. |
|  |  | What was done with the positioning data? | Positioning is a basic function of the smart deworming collar. By continuously collecting the movement trajectory of a single dog and uploading onto the RMS, combined with Big Data, we can draw movement maps for dog masses, to provide a reference for formulating the control strategy of echinococcosis, and we will analyze it in other manuscripts. |
|  | Reviewer #2 | the results are clearly and completely presented | Thank you very much for your encouragement and recognition of our work. |
| Conclusions | Reviewer #1: | How often do the collars need to be recharged? | The smart collar was charged (recharged) once a year. Embedded high-capacity rechargeable lithium batteries and low-power technology were employed to ensure that the collar was always powered. Once attached to dog, the electric energy is enough to keep the smart deworming collar run well for one year (12 delivery PQZ baits ), which saves a lot of manpower and makes it possible to transform the manual deworming mode to the smart deworming mode. Please see more details of the modification in the part of Discussion in revised manuscript. |
|  |  | The designation of part of this study as a “prospective cohort field study with random sampling” is a bit unclear. | Thank you very much for your suggestion, we revised “prospective cohort field study with random sampling” to "Randomized controlled study" |
|  |  | Please discuss study limitations as well as collar limitations. How expensive are the collars?  How do you plan to address non-compliance issues?  Do you see the bulk and weight of the collar being problematic? | Thank you very much for your professional suggestions. Study limitations as well as collar limitations are very important for not only further study but also optimization and upgrading of smart deworming collar. We have add the limitations in the part of Discussion of revised manuscript. The limitations of this study is its relatively short evaluation period, and the actual effect of implementation still needs to be evaluated for a longer time. The weight and size of the smart deworming collar limit its application to all dogs, further optimization and upgrading is urgent to reduce weight and size and make dogs more comfortable.  Health education for dog owners should be strengthened to improve their willingness (non-compliance issues) in attaching smart collars.. In addition, the associated economics and potential impact on dogs all need further to be evaluated. We will discuss the cost-effectiveness analysis of smart collars in other manuscript.  Yes, we noticed the bulk and weight of the collar in the original manuscript and modified these contents in Design of the deworming device of Methods in revised manuscript. |
|  | Reviewer #2: | the conclusions are vsupported by the data | Thank you very much for your encouragement and recognition of our work. |
| Editorial and Data Presentation Modifications? | Reviewer #1: | Based on the abstract, it is not clear what the smart collar actually does (i.e., the authors need to define “smart-collar deworming”). | Thank you very much for your professional suggestions. The smart deworming collar can deliver PZQ baits for dogs automatically, regularly, quantitatively with predominant characteristics of being waterproof, anti-collision, cold-proof and long life battery. We have add this information in the part Methods of Abstract in revised manuscript in accordance with your suggestions. |
|  |  | I don’t understand the use of the term “cross-species”. Perhaps just use the term zoonotic. | Thank you very much for your professional suggestions. We have revised the term “cross-species” to“ zoonotic” in the relevant parts in revised manuscript in accordance with your suggestions. |
|  |  | The authors indicate that the global burden of CE is approximately 1 million. One million what? | Thank you very much for your professional suggestions. We have add its unit “disability-adjusted life years (DALYs)” in the relevant parts in revised manuscript in accordance with your suggestions. |
|  |  | Dog deworming is likely to have little impact on the E. multilocularis wildlife cycle, which should be acknowledged. | Thank you very much for your professional suggestions. We agree with you very much. It is clear that dog deworming is likely to have little impact on the E. multilocularis wildlife cycle. However, domestic dogs (especially ownerless dogs and stray dogs) also play a certain role in the endemic (co-endemic) areas of alveolar echinococcosis. Therefore, the deworming of dogs still has public health significance. Please see the references 10, 11, 12. |
|  |  | If future versions of this paper are submitted, inclusion of page numbers would be helpful. | Thank you very much for your suggestion. We have add page numbers in revised manuscript. In addition, we also added the line numbers in revised manuscript. |
|  | Reviewer #2 | minor revisions | Thank you very much for your professional suggestions. |
| Summary and General Comments | Reviewer #1: | This is largely a proof-of-concept study looking at a dog collar that dispenses praziquantel baits. After reading the paper, I’m still not entirely sure how the collar works. Based on figure 1 (and the provided video), it appears that the collar is a smart pill dispenser that drops a praziquantel bait at predetermined intervals. However, the authors need to include additional information in the text about how the collar functions. | Thank you very much for good professional advice. We briefly describe the purpose and functions of the collar in the part of Design of the deworming device of Methods ( the smart deworming collar can deliver PZQ baits for dogs automatically, regularly, quantitatively with predominant characteristics of being waterproof, anti-collision, cold-proof and long life battery).  The steps for attaching the smart collar to the dogs were (1) matching the information of the dogs, the owners, and the smart collars and uploading the information to the RMS, (2) set deworming frequency such as once a month, once a quarter, even once every six months according to the local prevalence of echinococcosis, (3) set the PZQ baits dose for each dog according to the dog’s weight and size, (4) set deworming time (8:00 or 18:00 on the deworming date), and then the smart deworming collar will run according to the set procedure.  The advantages of employing the smart deworming collar for dogs included: (1) deworming automatically as the smart collar only needs to be attached to the dog once a year and will deliver bait containing PZQ 12 times automatically, which greatly saves originally planned manpower and cost; (2) deworming regularly; (3) deworming quantitatively and with conditions for each individual dog; (4) digital smart telemanagement and control as after the dog is attached a smart collar, the RMS will automatically identify and analyze the related information and remotely monitor the status of the collar in real time without having to enter the scene; (5) predominant characteristics of being waterproof, anti-collision, and cold-proof to ensure it runs well in the harsh climate.  We added the reference 25, in which we describes in details of the functions and advantages of the smart deworming collar and its installation and using steps. In general, the smart deworming collar attached to target dog for delivering PZQ baits automatically, regularly and quantitatively and was controlled by the RMS.  About the working principle of RMS and the smart deworming collar, please see the reference 26. |
|  | Reviewer #2: | The limitations of the use of the PZQ have been well developed in Larrieu and Zanini. It would be interesting to include some description of the substrate of the baits for the dogs to eat, given the bad smell and taste of PZQ | Thank you very much for good professional advice. As we know, ordinary praziquantel tablets have a low eating rate for canines due to its bitter taste and bad smell. The praziquantel chewable tablets (the PZQ baits) used in our study were prepared into microcapsules by embedding praziquantel, the favorite food (meat, fatty oil, etc.) of carnivores was selected as the core to develop an attractive core suitable for tablet production technology. This PZQ baits greatly not only conceals the bitter taste and unpleasant odor of praziquantel, but also has a good attractivity, so dogs and other carnivores can actively chew and/or swallow. We added the reference 28 in revised manuscript which describes more details about PZQ baits. |
|  |  | Although the manuscript is very detailed, it would be good to have some data on the variability of the weight of the dogs and the consequent estimation of the doses (1 or 2 pills) and consequently the estimation of the number of possible deworming. | Thank you very much for good professional advice. According to the exclusion criteria for dogs wearing collars, if dog weight is less than 5kg, dog age is less than 1 month, dog is pregnant, or dog is too small or too big, the dog is not suitable for wearing collars. According to the instructions of PZQ baits, in order to facilitate the administration, and to consider safety and effectiveness, the dosage of deworming for dog is: if dogs weight less than 20kg, 1 tablet per month (100mg); if dogs weight is 20kg or more, 2 tablets per month ( 200mg). We have add the information in the part of Interventions of revised manuscript. |
|  |  | A clearer description of the RMS and how deworming is activated would be helpful | Thank you very much for good professional advice. The RMS, which has the architecture of executive, sensing, network, analysis, and application layers, can realise a series of functions, including remote control, especially regular and automatic delivery of PZQ baits, data analysis, and result display.  The steps for attaching the smart collar to the dogs were (1) matching the information of the dogs, the owners, and the smart collars and uploading the information to the RMS, (2) set deworming frequency such as once a month, once a quarter, even once every six months according to the local prevalence of echinococcosis, (3) set the PZQ baits dose for each dog according to the dog’s weight and size, (4) set deworming time (6:00 AM on the deworming date), and then the smart deworming collar will run according to the set procedure.  The advantages of employing the smart deworming collar for dogs included: (1) deworming automatically as the smart collar only needs to be attached to the dog once a year and will deliver bait containing PZQ 12 times automatically, which greatly saves originally planned manpower and cost; (2) deworming regularly; (3) deworming quantitatively and with conditions for each individual dog; (4) digital smart telemanagement and control as after the dog is attached a smart collar, the RMS will automatically identify and analyze the related information and remotely monitor the status of the collar in real time without having to enter the scene; (5) predominant characteristics of being waterproof, anti-collision, and cold-proof to ensure it runs well in the harsh climate.  We briefly describe the purpose and functions of the collar in the part of Design of the deworming device of Methods and how it is used and we added the reference 25, which describes in details the functions and advantages of the smart deworming collar and its installation and using steps. When the smart deworming collar first attached and then all relevant information will be uploaded to the RMS automatically. About the working principle of RMS and the smart deworming collar, please see the reference 26. |
