# Supplementary material for: Smart deworming collar: A novel tool for reducing Echinococcus infection in dogs
Source: PLoS Negl Trop Dis. 2021 Jul 8;15(7):e0009443. doi: 10.1371/journal.pntd.0009443 (PMC8266112; doi:10.1371/journal.pntd.0009443)
Supplement: Attachment — Submitted filename: Responses to the review comments20210421.docx [file pntd.0009443.s003.docx]

Dear Editor,

Thank you very much for your sending the professional opinions and suggestions from the editors and reviewers.

According to your professional opinions, we performed revised version of our original manuscript, adding the measures to ensure the owners in the smart collar group recorded their dogs ingesting the PZQ baits at 6 a.m. on the deworming date, upgrading the reference 26 from preprint format to official publication format, correcting some inappropriate use of “rate” and replaced it with “proportion” (including them in the tables 3-5) and some other clerical errors，change the name of “Seni” to“ Seni district” and the name of “Hezuo” to “Hezuo city” in revised version (including them in the Tables 1, 3-6). A point-by-point response to the review comments as follows:

**Summary and General Comments**

Reviewer #1: "Thank you for clarifying most of my questions. My remaining comments are below.

**Question 1:** Please use care when using of the term “rate”. In most instances, I believe the authors mean “proportion”."

**Response:** Thank you very much for good professional advice. According to your suggestion, I reviewed the definition and usage about rate and proportion, I corrected some inappropriate use of “rate” and replaced them with “proportion” in revised manuscript (including them in the table 3-5) and we still look forward to your further advice.

**Question 2:** Please provide the actual sensitivity and specificity of the copro-ELISA rather than >90%.

**Response:** Thank you very much for good professional advice. According to your suggestion, we have upgraded the values of sensitivity and specificity (the sensitivity is 95.45% and the specificity is 96.97%). Here is the published reference for the copro-ELISA’s sensitivity and specificity, please see <https://kns.cnki.net/kcms/detail/detail.aspx?dbcode=CJFD&dbname=CJFDLAST2020&filename=HBYS202009007&v=BIIMyk7mNQe3Wkx%25mmd2BXJl9DgHydP2LteLDKVXplO0o4%25mmd2F2FryCt5n5rGQmy0Ov9bpEr>.

**Question 3:** It is still not clear if the information obtained from the manual deworming group (e.g., questions regarding whether the dog was dewormed and proof of deworming) was collected using a standardized questionnaire.

**Response:** Thank you very much for good professional advice. As stated in the **Methods** in manuscript, the subject of the questionnaire survey is the owner of the dog wearing the smart collar. That is to say, the dog owners in the control group (the manual deworming group) did not conduct a standardized questionnaire survey, but it was true that the relevant information, including dog information, dog owner information, and village information was collected standardized, the process and method were the same as the intervention group.

**Question 4:** I’m still a bit confused about how bait consumption was confirmed. I understand how this was conducted by the researchers upon initial attachment of the collar. However, could the authors please clarify how dog owners recorded whether or not a dog in the smart collar group ingested the praziquantel bait within 10 or 30 minutes? Were owners asked to observe the dogs at 6:00 a.m. on mornings when the bait was dispensed? Did the owners record this information in writing? Without a defined protocol, I’m a bit skeptical of these values.

**Response:** Thank you very much for good professional question. As stated in the part of **Interventions of Methods** in manuscript, delivery time of the bait was set to 6 a.m. on the deworming date. To ensure that the collected data of ingested the PZQ baits is true and effective, we had adopted many methods, including (1) at about 5:50 in the morning of the deworming day, the village dewormer (the village administrator) made a telephone call to owners remind them to observe and record whether their dogs swallowed the bait or not,(2) we had signed a letter of commitment with each dog owner in order to enhance their sense of compliance, (3) during the observation, the dog owner was asked to fill in **Record Form** about dog swallowing in time, take real-time photos, and send the photos to the village administrator within 30 minutes,(4 ) as an incentive(a reward), the local CDC will issue prizes to every dog owner who is qualified to complete the task. And as stated in the part of **Grouping and sample size of Field Trial of Methods** in manuscript, in Seni, 18 smart deworming collars were distributed in 14 villages of 12 townships, in Hezuo, a total of 523 smart deworming collars were distributed in 48 villages of 12 townships, So, to the dewormers (the village administrators), it is fully capable of accomplishing these things including calling the dog owners on the morning of the deworming day to remind them, to observe and take photos and record the information of dog swallowing. We have added these contents in part of **Interventions of Methods in** the revised manuscript.

**Question 5:** The text indicates that 741 dogs were sampled at baseline (line 269). However, table 1 indicates that only 687 dogs were sampled at time “0”. Since dogs were “missing” at all sampling time points, please clarify when the collars were placed relative to sample collection.

**Response:** Thank you very much for good professional question. According to the design, 741 dogs were sampled in the two groups in Seni and Hezuo, within a week before attaching the smart deworming collar to dogs, we collected dog feces samples in accordance with the requirements as baseline (at time “0”) , but 54 dogs were not obtained so we failed to collect their feces samples. We specifically evaluated this, especially in terms of statistics, the missing rate of 7.3% was considered acceptable and did not have a significant impact on the statistical results. The missing reason of dog is mainly not at home (settlement). Then we attached the smart deworming collar to each target dog according to the design, and the first feces discharged after being smart dewormed were collected, the time was within 1-2 days after each deworming day. As shown in Table 1, some dog feces samples were still missing in every batch, but it was all within the controllable range.

**Question 5:** Additional editing for language would be helpful. However, I will leave this to the Editor’s discretion.

**Response:** Thank you very much for your suggestions. We will try our best to improve the English writing level and polish the manuscript in strict accordance with the editor’s requirements.
